# Offal Chemical Composition from Veal, Beef, and Lamb Maintained in Organic Production Systems

**DOI:** 10.3390/ani9080489

**Published:** 2019-07-26

**Authors:** Wioletta Biel, Ewa Czerniawska-Piątkowska, Alicja Kowalczyk

**Affiliations:** 1 Department of Pig Breeding, Animal Nutrition and Food, the West Pomeranian University of Technology in Szczecin, Klemensa Janickiego 29, 71-270 Szczecin, Poland; 2 Department of Ruminant Science, the West Pomeranian University of Technology in Szczecin, Klemensa Janickiego 29, 71-270 Szczecin, Poland; 3 Department of Environment, Animal Hygiene, and Welfare, Wrocław University of Environmental and Life Sciences, Chełmońskiego 38C, 51-630 Wrocław, Poland

**Keywords:** veal, beef, lamb, *musculus semitendinosus*, liver, heart, kidney, tongue, brain, proximate composition, macro-, microelements, energy value

## Abstract

**Simple Summary:**

Almost all byproducts are a rich source of trace elements, whose levels/amounts are usually much higher in byproducts such as offal than in muscular tissues. Furthermore, the demand for offal is likely to increase with the rapidly growing population, because it can be commercially sold for human and animal nutrition and is economically advantageous (e.g., high profitability). Therefore, further research into consumer perception of offal consumption is needed, as well as investigations of the potential use of the nutrients contained in offal. Such studies will ensure a comprehensive presentation of the factors that affect the demand for offal.

**Abstract:**

The aim of the study is to compare the content of nutrients, including selected macro- and micro-elements, in *musculus semitendinosus* and offal (liver, heart, kidneys, tongue, brain) derived from animals (calves, beef cattle, and lambs) that are fed and maintained in organic production conditions. The experimental material consisted of 60 animals: 20 calves, 20 beef cattle, and 20 lambs. This research was carried out using Limousin cattle and Ile de France sheep. From the obtained results, it is concluded that the physicochemical and nutrient composition varied significantly among the organs and species studied. Almost all byproducts are a rich source of trace elements, whose levels/amounts are usually much higher in byproducts such as offal than in muscular tissues. Also, for economic reasons (profitability), byproducts (offal) can be commercially sold for human and animal nutrition. Byproducts are processed and incorporated into many food products and provide competitive nutritional value for use by tissues and muscles (vitamins and elements).

## 1. Introduction

The definition of “meat” in Council Regulation (EC) No 700/2007 of 11 June 2007 also includes offal. A specific feature of meat and offal is their nutritive elements, including high-quality protein, fat, and mineral and vitamin content, which largely determine taste and dietary value. For example, the liver has a high nutritional value. According to Florek et al. [1], the levels of certain minerals, such as iron, zinc, magnesium, and calcium, are higher in veal and beef liver than in muscular tissue. Offal with favorable composition, minerals, vitamins also includes the brain, tongue, heart, and kidneys. The spleen and lungs are consumed less and have a lower commercial value as a result of their histological structure. The quality of raw materials obtained from ruminants is influenced not only by genetic factors but also, to a large extent, by environmental factors (including animal nutrition). Since the 1980s, there has been systematic and intensive development of organic farming, both in Poland and worldwide, and growing demand for organic food. An increasing number of farms are transforming their operations into organic production. On farms, strictly defined production methods are applied, controlled by independent, professional organizations that provide certifications. The consumers involved in free trade have high expectations for organic products. The nutritional value of meat is one of the evaluated aspects during assessments of organic food quality. However, the current organic animal production in the European Union countries is small, amounting to about 2% of the total production of cow’s milk and beef cattle [2].

The aim of this work is to compare the content of basic nutrients, including selected macro- and micro-elements, of *musculus semitendinosus* and offal (liver, heart, kidneys, tongue, brain) derived from calves, beef cattle, and lambs that are managed and fed in organic production conditions.

## 2. Materials and Methods

### 2.1. Animals

All animals used in the experiment came from production flocks. All activities related to their maintenance and slaughter were routinely carried out in production conditions. The consent of the ethics committee was not required for this experiment.

The experimental material comprised a total of 60 animals: 20 calves, 20 beef cattle, and 20 lambs. The research was carried out on Limousin cattle and Ile De France sheep from herds kept in the Western Pomerania voivodeship and maintained in organic production systems. The energy and protein supply and requirements were derived according to Institut National de la Recherche Agronomique (INRA) [3]. Animal feed was obtained from certified organic crops with an additional mineral mixture that is authorized for use on organic farms. After reaching the age of 120 days, the lambs were slaughtered (the minimum weight of the carcass was 24 kg), as well as 20 randomly selected cattle. The calves were slaughtered at the age of 3 months with a weight of approx. 95 kg ± 3.5 kg. The average weight of beef cattle at slaughter was 575.3 kg ± 13.6 kg. The animals were delivered to the slaughterhouse and slaughtered in accordance with Council Regulation [4].

### 2.2. Sample Preparation

Afterwards, the carcass and offal of individual animals were inspected and deemed acceptable for human consumption and the heart, kidneys, tongue, liver, and brain were collected from the slaughterhouse. *Musculus semitendinosus* and the five above-listed organs were removed from the animals. Each organ was individually labeled, frozen, and transported to the West Pomeranian University of Technology for further analyses. At 48 h post-mortem, samples for chemical analyses were homogenized using a Büchi Mixer B-400 (Büchi Labortechnik, Flawil, AG, Switzerland). Samples were analyzed in duplicate.

### 2.3. Proximate Composition

The moisture content (%) of all samples was determined according to the Association of Official Analytical Chemist’s Standard Methods [5]. The moisture content was determined by drying a 2 g sample at 100–105 °C for 24 h. After drying the samples, they were weighed and ashed at 500 °C for 6 h to determine the crude ash content. The fat content was determined by homogenizing the samples in a blender, followed by diethyl ether extraction. The protein content (N × 6.25) was determined by the Kjeldahl method using a Büchi B-324 distillation unit (Büchi Labortechnik, Flawil, AG, Switzerland), the sample residue from the fat analysis, and a mortar to generate a fine powder.

### 2.4. Mineral Analysis

The concentration of phosphorus was determined by the Egner–Riehm colorimetric method with ammonium molybdate and a wavelength 660 nm using a Specol 221 apparatus (866287, Carl Zeiss Jena, Jena, Germany). An Atomic Absorption Spectrometer (ASA) apparatus (iCE 3000 Series, Thermo Fisher Scientific, Waltham, MA., USA) was used to measure calcium, potassium, and sodium by emission flame spectroscopy, and absorption flame spectroscopy was used to measure magnesium, iron, zinc, copper, manganese, lead, and cadmium. The material for macro-component concentration was dissolved in concentrated sulfuric acid (H_2_SO_4_) and perchloric acid (HClO_4_), while the material for micro-component concentration was dissolved in a mixture of nitric acid (HNO_3_) and perchloric acid (HClO_4_). Before assaying the Ca content, K and Mg trials were appropriately diluted. Other mineral compounds were determined in concentrated samples. Selenium was determined using nuclear spectrometry (ASA: Atomic Absorption Spectrometry) with electrothermal atomization in graphite cuvettes (ET-AAS) on Hitachi’s Z-5000 camera (Japan) with Zeeman background correction.

### 2.5. Energy Values

Energy values were calculated from the content of crude protein and fat. The computations were based on the physiological (Atwater) energy equivalents (4.0 kcal for protein, and 9.0 kcal for fat) and expressed as kcal per 100 g of wet tissue.

### 2.6. Statistical Analyses

The obtained results were statistically evaluated, and the average and standard deviation of individual features were calculated. Significant differences were calculated by one-way analysis of variance using the Duncan multiple range test with the Statistica^®^ (Hamburg, Germany) 12.5 PL program [6]. 

The linear model is as follows:

Yij = μ + ti + eij(1)

where Yij is the variable being analyzed (basic nutrients, macro- and micro-elements of *musculus* semitendinosus and offal), μ is the expected value, ti is an experimental factor (lambs, calves, beef cattle), and eij is a random error effect.

## 3. Results

The average values of the chemical composition of calf, beef, and lamb organs are given in Table 1, Table 2 and Table 3.

Table 1 presents the average values of the chemical composition and energy of the materials tested. There were no significant differences in humidity between the type of offal and meat and individual organs. The average moisture content in the tongue of beef was significantly higher (*p* < 0.01) than that of lamb by 10.5%. The highest average moisture, 80.0%, was recorded for calf brain.

The highest protein content was detected in *musculus semitendinosus* from beef cattle (20.71%), and it was significantly different (*p* < 0.05) from the average value detected in *musculus semitendinosus* from veal (17.47%) and lamb (18.25%). High average values of protein were found in whole lamb liver (20.40%) and beef liver (20.30%). Moreover, significant differences (*p* < 0.01) in protein content was found in the kidneys of all three types of meat. Beef tongues were characterized by a significantly higher (*p* < 0.01) protein content (19.00%) than lamb tongues (15.50%). In the remaining organs, there were no significant differences in protein content among the individual types of meat. The lowest protein content was recorded for calf brain (10.15%).

The average fat content in *musculus semitendinosus* from lamb was 15.15% and 2% lower than the average fat content observed in the tongues. The fat content in lamb tongues was significantly higher (*p* < 0.01) than that in beef or veal offal (11.4% and 11.7%). Significant differences (*p* < 0.01) in fat were also observed between lamb *musculus semitendinosus* (15.15%) and beef *musculus semitendinosus* (12.14%). The lowest fat content was recorded in lamb kidneys (2.85%). The average ash content in the individual organs of different types of meat did not significantly differ. The highest value was recorded in lamb liver (1.40%), and the lowest content was found in lamb *musculus semitendinosus* (0.70%).

The highest average energy content was recorded in beef tongues (224.0 kcal), which was significantly higher (*p* < 0.01) than the values obtained for lamb and calf offal by 4 and 94 kcal, respectively. Lamb hearts were significantly (*p* < 0.05) higher in energy content than calf hearts (by 10 kcal). On the other hand, the lowest energy content was recorded for beef kidneys: The average energy content was 13 kcal and significantly lower (*p* < 0.01) than that contained in lamb kidneys (97 kcal) and calf kidneys (98 kcal).

Table 2 presents the average values of macronutrients in the organs tested. The highest content of calcium (Ca) was observed in the bovine brain (43.0 mg/100 g), and it was significantly higher (*p* < 0.01) than the average value of offal from calves (33.0 mg/100 g) and lamb (34.5 mg/100 g). In the beef *musculus semitendinosus*, hearts, and kidneys, a significantly higher (*p* < 0.01) Ca content was observed compared with calf organs. The lowest Ca content was recorded for calf and beef liver (5.0 mg/100 g) and veal calf hearts (5.0 mg/100 g). Significant differences (*p* < 0.01) in phosphorus content (P) were observed between the hearts and tongues of lamb and beef. Beef hearts had significantly (*p* < 0.01) more P (by 42.0 mg/100 g) than lamb. For tongues, lamb was characterized by a significantly higher (*p* < 0.01) content of this macro-element (185.0 mg/100 g) compared with beef (133.0 mg/100 g). Significantly higher content of P (*p* < 0.01) was also observed in beef leg compared with veal calf *musculus semitendinosus* (by 25.0 mg/100 g). Beef liver was characterized by the highest content of this macro-element and has an average P content of 387.0 g/100 g, whereas the lowest P content was recorded for beef tongues (133.0 g/100 g). The highest sodium content (Na) was observed in beef kidneys (185.0 mg/100 g), and the content significantly differed (*p* < 0.01) from the mean value observed in lamb kidneys by 30 mg/100 g. Equally high Na content was observed in the brain (veal calves, beef, and lamb). The liver, heart, and leg contained almost half of the Na content compared with the Na in kidneys or brain. 

Potassium (K) content dominated in calf hearts and was more than twice the content in beef and lamb. Significant differences (*p* < 0.01) in K content were observed between calf tongues (82.0 mg/100 g) and beef tongues (69.0 mg/100 g), as well as veal calf brains (312.0 mg/100 g) and beef brains (274.0 mg/100 g).

No significant differences in Mg content were found in the tested samples. The highest value was recorded in beef *musculus semitendinosus* (24.0 mg/100 g). Veal calf liver, beef hearts, and *musculus semitendinosus* of lamb were characterized by the same Mg content (20.0 mg/100 g). The lowest Mg content was recorded for lamb brain (12.0 mg/100 g).

Table 3 presents the average values of micronutrients in the tested organs. Significant differences (*p* < 0.01) in the iron content (Fe) were observed between lamb liver (7.25 mg/100 g) and beef liver (4.8 mg/100 g) and between *musculus semitendinosus* from beef (1.8 mg/100 g) and that from lamb (1.34 mg/100 g). The Fe content in the bovine brain was significantly (*p* < 0.05) higher than that in lamb brain (by 0.9 mg/100 g).

The highest zinc (Zn) content was recorded in the veal calf liver (12.0 mg/100 g), and this value was significantly different (*p* < 0.01) from the values obtained from lamb and beef liver. Furthermore, the content of Zn in calf liver was almost three times higher than that in lamb or beef. Compared with the calf rim, a significantly lower (*p* < 0.05) Zn content was observed in the leg of lamb (by 2.87 mg/100 g). By far, the lowest content of Zn was found in the brain (veal, lamb, and beef): It was more than ten times lower than the Zn content in veal calf liver.

The highest results for copper (Cu) content were obtained from the liver. Its value in veal was 11.77 mg/100 g and significantly different (*p* < 0.05) from the value obtained from lamb (6.88 mg/100 g). Lamb tongues were significantly (*p* < 0.05) higher in Cu compared with Cu content in beef (by an average of 0.13 mg/100 g). There were no significant differences in the content of this micronutrient among the remaining organs examined. 

The highest content of manganese (Mn) was found in veal calf hearts (0.35 mg/100 g) and beef hearts (0.29 mg/100 g). The obtained results were significantly higher (*p* < 0.01) than those obtained from lamb by about three-fold. The content of Mn was also significantly different (*p* < 0.05) in the kidneys (beef, lamb, and veal). The lowest content of Mn was recorded for calf kidneys (0.01 mg/100 g). A high content of selenium (Se) was recorded for the kidneys and liver (beef, veal, lamb). In the *musculus semitendinosus*, the tongues, kidneys, and liver, significant differences were found (*p* < 0.01) in the content of this microelement The lowest content of Se was obtained from the *musculus semitendinosus* of lamb, with a value of only 4.5 µg/100 g, which is almost thirty times lower than the Se content in beef kidneys (139.0 µg/100 g). No toxic elements (Pb, Cd) were found in the tested samples of meat.

## 4. Discussion

Meat and meat products are an essential component of the human diet because they provide essential nutrients [7]. During the process of transforming livestock into meat in slaughterhouses, many different products are generally prepared, and they can be used by humans as food or processed as secondary products [8]. Also, they can be used to reduce the risk of malnutrition, especially in developing countries, where food insecurity and climate change are a significant problem [9] that has led to a deficiency in critical nutrients, such as vitamins and minerals, in human populations [10]. It has been proven that offal contains essential nutrients, such as vitamins (B1, B2, B6, and folic acid), proteins, minerals, and fat, as well as essential polyunsaturated fats and amino acids, whose contents are comparable to those found in muscle tissue (Ockerman and Basu) [11].

The liver is 1–2% of the live weight of cattle and is an important, edible organ that is richer in minerals and vitamins than other tissue and muscle [12]. Compared with the values reported by other researchers [12,13,14], a lower moisture content in beef, calf, and lamb liver was observed, as well as higher protein and ash content and similar fat and energy content [12]. The contents of macro- and micro-nutrients in the liver reported in this study are similar to the values reported for livers of different species [1,15,16,17,18]. Florek et al. [1] showed that the content of K, Zn, and Fe in liver was competitive with that in other organs, which was also observed in our study.

The moisture of hearts, kidneys, tongues, and brains ranged from 66.5% to 80.0% and was consistent with the values obtained by other researchers [1,19]. The protein content ranged from 10.15% in veal calf brain to 19% in beef tongues. Ockerman and Basu presented similar values in their research [11]. Among the byproducts tested, the highest fat content was recorded for the bovine brain (10.30%), which corresponds to the results obtained by other researchers [11,20]. The brains were characterized by a low content of micronutrients, including the lowest Zn content in all the organs examined. Similar values were given by Nollet and Toldra [21]. Zinc is an important element in human nutrition, and its deficiency can have many health consequences [22,23,24]. In our study, it was observed that offal, such as liver, kidneys, and tongues, contains enough of this element to counteract deficiencies. 

Noteworthy is the nutritional value of the tongues determined in this research. They are characterized not only by high protein content (19%) but also by fat (17.15%) and the highest energy value among the examined tissues (224 kcal). Various studies have reported that it provides high-quality proteins, vitamins, and minerals that may be nutritionally beneficial [25,26]. They are rich in iron, zinc, choline, and vitamin B12, and it has been shown that any portion of boiled bovine tongue can increase the iron content in the body and provide about 28% and 12% of the recommended daily iron intake for men and women, respectively [26].

The *musculus semitendinosus* had the lowest moisture value among the examined organs (66–67%). In terms of protein content, its value was not higher than the protein in liver (20.40%), but it was significantly higher than that in brain by about 7–9%. The protein content corresponded to that reported by Ockerman and Basu [11]. The fat content obtained in the tests was high, ranging from 12.14% to 15.15%. The ash content was the lowest in comparison with other tested organs. Other studies have also reported higher ash content in calf and sheep liver, kidneys, hearts, lungs, and spleens [1,15]. In terms of energy, the leg had values ranging from 198 to 215 kcal. Our results show that the content of macro- and micro-elements varied significantly among the examined organs. The Fe content was the lowest in *musculus semitendinosus* (from 0.53 to 1.34 mg/100 g), and low values were also obtained by analyzing this organ for Cu, Mn, and Se content. Murphy and Allen [27] showed that insufficient intake of Fe and Zn causes anemia, rickets, and cognitive impairment in humans. Also, minerals such as Se, Cu, Zn, Fe, and Mn are critical for enzymes that function to counteract free radicals in the body. This research showed that, compared with skeletal muscles (thigh), organ meats had higher concentrations of Zn, Fe, Cu, Mn, Ca, and Na, which has also been confirmed by other authors [28]. Iron, manganese, zinc, and copper are elements that are necessary for maintaining human health, and their low consumption can lead to symptoms of nutrient deficiency [29].

## 5. Conclusions

From the obtained results, it is concluded that the physicochemical and nutrient composition varied significantly among the organs and species studied. Almost all byproducts are a rich source of trace elements, whose levels/amounts are usually much higher in offal than in muscular tissues (*musculus semitendinosus*). In addition, byproducts (offal) are beneficial for economic reasons (profitability) and can be commercially sold for human and animal nutrition. They are processed and incorporated into many food products and have competitive nutritional value (vitamins, micro- and macro-elements) for tissues and muscles. Furthermore, the demand for organs is likely to increase as the population continues to grow rapidly. Therefore, further research should be carried out regarding the perception of offal consumption by consumers, as well as the feasibility of using nutrients contained in offal. This will ensure a comprehensive presentation of factors that affect the demand for offal.

## Figures and Tables

**Table 1:** Chemical composition (% of wet tissue) and energy value (kcal/100 g) of the tested organs (mean ± s.e.).

Item	Moisture			Protein			Fat			Ash			Energy		
	Calves	Beef	Lamb	Calves	Beef	Lamb	Calves	Beef	Lamb	Calves	Beef	Lamb	Calves	Beef	Lamb
Liver	70.59 ± 0.06	70.00 ± 0.53	71.00 ± 0.47	19.36 ± 0.66	20.30 ± 0.28	20.40 ± 0.38	4.75 ± 0.23	3.60 ± 0.92	5.20 ± 0.68	1.35 ± 0.14	1.30 ± 0.19	1.40 ± 0.09	140 ± 1.47	136 ± 2.67	140 ± 1.33
Heart	77.00 ± 0.33	77.00 ± 0.33	76.00 ± 0.67	17.15 ± 0.26	17.25 ± 0.36	16.27 ± 0.62	3.95 ± 0.58	3.95 ± 0.58	5.68 ± 1.15	1.10 ± 0.07	1.00 ± 0.17	1.10 ± 0.08	110 ^b^ ± 4.00	112 ± 2.00	120 ^a^ ± 6.00
Kidney	79.00 ± 7.88	77.00 ± 9.17	79.00 ± 7.17	15.70 ^B^ ± 0.53	17.50 ^A^ ± 1.27	15.50 ^C^ ± 0.73	3.12 ± 0.10	3.10 ± 0.08	2.85 ± 0.17	1.10 ± 0.22	1.20 ± 0.12	1.30 ± 0.02	98 ^B^ ± 28.67	13 ^A^ ± 56.33	97 ^C^ ± 27.67
Tongue	74.50 ± 2.17	77.00 ^A^ ± 9.17	66.50 ^B^ ± 5.83	17.15 ± 0.07	19.00 ^A^ ± 0.78	15.50 ^B^ ± 1.27	5.45C ± 4.00	5.75 ^B^ ± 3.70	17.15 ^A^ ± 7.70	1.00 ± 0.03	1.19 ± 0.16	0.90 ± 0.13	130 ^C^ ± 61.33	224 ^A^ ± 32.67	220 ^B^ ±28.67
Brain	80.00 ± 1.4	76.29 ± 2.31	79.50 ± 0.90	10.15 ± 1.40	10.86 ± 0.69	10.50 ± 1.05	8.15 ± 0.86	10.30 ± 1.29	8.58 ± 0.43	1.30 ± 0.02	1.10 ± 0.22	1.20 ± 0.12	115 ± 11.00	143 ± 17.00	120 ± 6.00
*Musculus semitendinosus*	67.67 ± 1.00	66.00 ± 0.67	66.35 ± 0.32	17.47 ^c^ ± 1.34	20.71 ^a^ ± 1.90	18.25 ^b^ ± 0.56	14.75 ± 0.74	12.14 ^B^ ± 1.87	15.15 ^A^ ± 1.14	0.90 ± 0.09	1.10 ± 0.11	0.70 ± 0.29	207 ± 0.50	198 ± 8.67	215 ± 8.33

The mean values in the rows marked with different letters differed significantly. ^a,b,c^ *p* ≤ 0.05; ^A,B,C^ *p* ≤ 0.01.

**Table 2:** Macronutrients in the tested organs (mg/100 g).

Item	Ca			P			Na			K			Mg		
	Calves	Beef	Lamb	Calves	Beef	Lamb	Calves	Beef	Lamb	Calves	Beef	Lamb	Calves	Beef	Lamb
Liver	5.00 ^B^ ± 0.73	5.00 ^B^ ± 0.67	7.00 ^A^ ± 1.33	375.00 ± 0.67	387.00 ± 11.33	365.00 ± 10.67	75.00 ± 2.67	68.00 ± 3.33	71.00 ± 0.33	310.00 ± 1.33	312.00 ± 0.67	312.00 ± 0.67	20.00 ± 0.99	18.00 ± 2.90	19.00 ± 1.99
Heart	5.00 ^B^ ± 1.33	8.00 ^A^ ± 1.67	6.00 ± 0.33	211.00 ± 9.33	218.00 ^A^ ± 16.33	176.00 ^B^ ± 25.67	75.00 ^b^ ± 12.00	97.00 ^a^ ± 10.00	89.00 ± 2.00	761.00 ^A^ ± 20.70	285.00 ^C^ ± 16.90	316.00 ^B^ ± 13.80	18.00 ± 0.33	20.00 ± 1.67	17.00 ± 1.33
Kidney	10.00 ^b^ ± 3.20	13.00 ^a^ ± 0.22	13.00 ^a^ ± 0.20	240.00 ± 6.67	255.00 ± 8.33	245.00 ± 1.67	175.00 ± 3.30	185.00 ^A^ ± 13.33	155.00 ^B^ ± 16.67	272.00 ± 2.00	263.00 ± 7.00	275.00 ± 5.00	16.00 ± 0.67	17.00 ± 0.37	17.00 ± 0.33
Tongue	7.00 ± 0.46	6.39 ± 1.07	9.00 ± 1.54	155.00 ± 2.67	133.00 ^B^ ± 24.57	185.00 ^A^ ± 27.33	82.00 ^A^ ± 6.67	69.00 ^B^ ± 6.33	75.00 ± 0.33	270.00 ^B^ ± 10.67	315.00^A^ ± 34.33	257.00 ± 23.67	15.00 ± 2.33	16.00 ± 1.33	21.00 ± 3.67
Brain	10.00 ^B^ ± 10.50	43.00 ^A^ ± 22.50	8.50 ^C^ ± 12.00	272.00 ± 27.67	362.00 ± 62.33	265.00 ± 34.67	125.00 ± 3.00	126.00 ± 4.00	115.00 ± 7.00	312.00 ^A^ ± 18.33	274.00 ^B^ ± 19.67	295.00 ± 1.33	14.00 ± 1.00	13.00 ± 0.00	12.00 ± 1.00
*Musculus semitendinosus*	7.00 ^Bba^ ± 4.00	16.00 ^Aa^ ± 5.00	10.00 ^Aa^ ± 1.00	170.00 ^A^ ± 10.00	195.00 ^B^ ± 15.00	175.00 ^AB^ ± 5.00	70.00 ^A^ ± 4.67	53.00 ^B^ ± 12.33	73.00 ± 7.67	285.00 ± 12.47	319.00 ± 22.67	285.00 ± 11.33	18.00 ± 2.67	24.00 ± 3.33	20.00 ± 0.67

The mean values in the rows marked with different letters differ significantly. ^a,b,c^ *p* ≤ 0.05; ^A,B,C^ *p* ≤ 0.01.

**Table 3:** Micronutrients in the tested organs (mg/100 g).

Item	Fe			Zn			Cu			Mn			Se [µg]		
	Calves	Beef	Lamb	Calves	Beef	Lamb	Calves	Beef	Lamb	Calves	Beef	Lamb	Calves	Beef	Lamb
Liver	6.40 ± 0.25	4.80 ^B^ ± 1.35	7.25 ^A^ ± 1.10	12.00 ^AB^ ± 1.72	4.00 ^C^ ±2.85	4.55^B^ ±2.30	11.77 ^a^ ± 2.33	9.66 ± 0.22	6.88 ^b^ ± 2.56	0.260 ± 0.02	0.290 ± 0.05	0.182 ± 0.06	22.6 ^C^ ± 6.32	39.00 ^B^ ± 2.22	82.00 ^A^ ±8.53
Heart	4.25 ± 0.13	4.30 ± 0.08	4.06 ± 0.22	1.27 ±0.31	1.70 ±0.12	1.77 ±0.19	0.350 ± 0.018	0.395 ± 0.030	0.350 ± 0.015	0.350 ^A^ ± 0.008	0.290 ^B^ ± 0.05	0.046 ^C^ ± 0.004	32.00 ± 3.33	21.00 ± 7.67	33.00 ± 4.33
Kidney	3.40 ± 1.38	4.60 ± 0.18	6.35 ± 1.57	1.95 ±0.34	1.95 ±0.10	2.25 ±0.20	0.495 ± 0.035	0.430 ± 0.030	0.456 ± 0.004	0.070 ^c^ ± 0.04	0.140 ^a^ ± 0.03	0.115 ^b^ ± 0.01	80.00 ^C^ ± 34.67	139.00 ^A^ ± 24.33	125.00 ^B^ ± 10.33
Tongue	2.70 ± 0.22	2.15 ± 0.33	2.60 ± 0.12	2.65 ±0.21	2.32 ±0.12	2.35 ±0.09	0.150 ± 0.01	0.070 ^b^ ±0.07	0.200 ^a^ ±0.06	0.025 ± 0.003	0.015 ± 0.013	0.045 ± 0.017	6.00 ^C^ ± 2.50	12.00 ^A^ ± 1.00	15.00 ^B^ ± 4.00
Brain	2.15 ± 0.03	2.55 ^a^ ± 0.43	1.65 ^b^ ± 0.47	1.15 ±0.06	1.02 ±0.20	1.15 ±0.07	0.230 ± 0.01	0.200 ± 0.03	0.250 ± 0.02	0.035 ± 0.009	0.040 ± 0.004	0.045 ± 0.001	10.00 ± 1.15	7.55 ± 1.30	9.00 ± 0.15
*Musculus semitendinosus*	0.53 ± 0.35	1.80 ^A^ ± 0.58	1.34 ^B^ ±0.12	2.35 ^b^ ±1.34	3.50 ±0.19	5.22^a^ ±1.53	0.095 ± 0.025	0.091 ± 0.029	0.175 ± 0.055	0.010 ± 0.004	0.012 ± 0.002	0.021 ± 0.07	6.50 ^B^ ± 5.17	24.00 ^A^ ± 2.33	4.50 ^C^ ± 7.17

The mean values in the rows marked with different letters differ significantly. ^a,b^ *p* ≤ 0.05; ^A,B,C^ *p* ≤ 0.01.
